# Report on the rare disease consortium Japan inaugural symposium - July 18, 2023, shonan health innovation park, Japan

**DOI:** 10.1177/22143602251356742

**Published:** 2025-07-22

**Authors:** Toshifumi Yokota, Naoto Inukai, Hiroyuki Shibasaki, Harumasa Nakamura, Shinnichi Yokota, Yoshitaka Nakamura, Rikako Yamauchi, Keiko Ishigami, Akitsu Hotta, Hideo Miki, Yoshitsugu Aoki

**Affiliations:** 1 Department of Medical Genetics, Faculty of Medicine and Dentistry, 3158 University of Alberta, Edmonton, AB, Canada; 2 R&D Research, Target Validation Sciences, Takeda Pharmaceutical Company Limited, Kanagawa, Japan; 3 Japan Muscular Dystrophy Association, Tokyo, Japan; 4 Department of Clinical Research Support, 26353 National Center of Neurology and Psychiatry, Tokyo, Japan; 5 Laboratory for Medicinal Chemistry Research, 13350 Shionogi & Co., Ltd., Osaka, Japan; 6 Discovery Research Laboratories in Tsukuba, Nippon Shinyaku Co., Ltd, Tsukuba, Ibaraki, Japan; 7 Strategic Alliance Office, 13593 RIKEN, Saitama, Japan; 8 Department of Commercial and Business Development, iPark institute Co., Ltd., Kanagawa, Japan; 9 Center for iPS cell Research and Application, 12918 Kyoto University, Kyoto, Japan; 10 Drug Discovery Strategy and Planning Office, C4U Corporation, Tokyo, Japan; 11 Department of Molecular Therapy, National Institute of Neuroscience, 26353 National Center of Neurology and Psychiatry, Kodaira, Tokyo, Japan

**Keywords:** rare diseases, antisense oligonucleotides, patient-centered care, health policy, orphan drug production, biopharmaceutics, genome-wide association study, patient advocacy, translational research

## Abstract

The Rare Disease Consortium Japan (RDCJ) is a newly formalized cross-sector initiative launched to address the urgent and growing needs of individuals living with rare diseases in Japan, aiming to support a wide range of rare conditions, including—but not limited to—neuromuscular diseases. RDCJ hosted its inaugural symposium on July 18, 2023, at the Shonan Health Innovation Park. This symposium aimed to address the unique challenges of rare diseases through a collaborative approach involving industry, government, academia, patients, and the community. The event brought together these stakeholders to discuss the current state and future directions of rare disease research and treatment in Japan. The event featured a range of speakers and panel discussions on the state of drug development, patient journeys, and the future of patient-centered healthcare services. Highlights included a keynote on antisense oligonucleotide therapeutics and sessions on patient-centered healthcare, human-centered design in healthcare, and innovative approaches to rare disease treatment. This report provides a detailed overview of the symposium, the key takeaways from each session, and the future directions for RDCJ.

## Introduction

Rare diseases, also known as orphan diseases, encompass a wide variety of conditions that often lead to chronic health issues, disability, and a significant impact on the quality of life for patients and their families.^1^ Despite the individual rarity of these diseases, collectively they affect millions of people worldwide.^2^ These conditions, defined by the World Health Organization as affecting fewer than 1 in 2000 people per disease, encompass over 8000 different disorders globally, impacting more than 350 million individuals.^3^ Despite significant socio-economic burdens, over 90% of these diseases lack effective treatments.^4^ In Japan, the need for a concerted effort to tackle rare diseases has become increasingly apparent, driven by the challenges faced by patients in accessing proper diagnosis, care, and effective treatments.^5^

Rare Disease Consortium Japan (RDCJ) was established with a mission to address these challenges through a collaborative approach. The consortium aims to create a sustainable ecosystem for drug development and patient support by fostering partnerships among academia, industry, government, and patient advocacy groups. This approach not only aims to expedite the development of new treatments but also to ensure that patients receive comprehensive and compassionate care. The RDCJ's vision includes enhancing regulatory frameworks, improving patient registries, and establishing robust clinical trial networks to accelerate the discovery and delivery of innovative therapies. To reflect the evolution of RDCJ, it is also important to note that the consortium was formally established as a legal entity on February 29, 2024—Rare Disease Day. This milestone marked a new chapter in its development, enabling greater structural coordination and advocacy. Since the symposium, RDCJ has launched a national “Physician Needs Survey” to identify clinical challenges, strengthened collaborations with advocacy groups such as ASrid (Advocacy Service for Rare and Intractable Diseases) and industry consortia, and initiated preparations for a follow-up symposium in 2025 and 2026.

Historically in Japan, the term “Nanbyo” (Intractable Disease) has been used to categorize conditions qualifying for government support based on criteria such as disease severity, rarity, and treatment availability. However, this designation does not always align with the globally accepted definition of “Rare Disease,” which is typically based on disease prevalence. While there is substantial overlap between the two categories, the shift in recent years towards using “Rare Disease” reflects an effort to harmonize with international standards, particularly those upheld by global research networks such as the International Rare Diseases Research Consortium (iRDiRC). The RDCJ represents part of this alignment, seeking to integrate Japan's unique healthcare system with international frameworks for rare disease research and advocacy. As part of this effort, RDCJ shares synergistic goals with iRDiRC and benefits from the participation of its members in international policy-shaping initiatives, including Dr. Yoshitsugu Aoki's contribution to the iRDiRC Rare Disease Funding Models Task Force^6^ and Dr. Harumasa Nakamura's in Clinical Research Networks Task Force for Rare Diseases.^7^

The RDCJ held its inaugural symposium on July 18, 2023, at the Shonan Health Innovation Park in Fujisawa, Japan. It featured a range of speakers and panel discussions on the state of drug development, patient journeys, and the future of patient-centered healthcare services. Highlights of the event included a keynote on antisense oligonucleotide therapeutics, sessions on patient-centered healthcare, human-centered design in healthcare, and innovative approaches to rare disease treatment. The event was a significant milestone in the efforts to address the challenges associated with rare diseases in Japan. The symposium brought together various stakeholders, including representatives from industry, government, academia, patient groups, and the broader community, to discuss and collaborate on strategies to improve the diagnosis, treatment, and overall care for individuals with rare diseases.

## Opening remarks

### Greeting by Dr. Toshio Fujimoto

Dr. Toshio Fujimoto, President and CEO of iPark Institute Co., Ltd.,., opened the symposium with a heartfelt story from his days of medical residency, underscoring the importance of empathy and understanding in the treatment of rare disease patients. He recounted his encounter with a patient suffering from Kartagener syndrome at a university hospital, a meeting that profoundly impacted his perspective on patient care.^8^ During his studies in Germany, Dr. Fujimoto received a meticulously crafted embroidered gift and a heartfelt letter from her, which highlighted her gratitude and resilience.

Reflecting on this experience, Dr. Fujimoto admitted his initial ignorance of the patient's ability to create such intricate embroidery due to his preconceived notions about those with rare diseases. This moment of realization led him to cherish the annual tradition of sending Christmas cards to her, a practice he maintained until her death a couple of years afterwards.

Dr. Fujimoto highlighted the stark reality faced by individuals with rare diseases, noting that there are approximately 8000 rare diseases globally, with over 90% still lacking effective treatments.^3^ He emphasized the pressing need to improve the environment for these patients, who continue to face significant challenges. Calling for collective action, he expressed his aspiration to create a society where individuals with rare diseases can live comfortably and contribute meaningfully. He outlined the mission of RDCJ to establish a sustainable ecosystem for drug development and patient support, inviting all attendees to join in this vital endeavor.

## Vision and mission of RDC Japan

### Presentation by Dr. Yoshitsugu Aoki

Dr. Yoshitsugu Aoki, Head of the Department of Molecular Therapy at the National Institute of Neuroscience, NCNP, took the stage next, representing the RDCJ's Preparatory Committee. Dr. Aoki shared the foundational experiences that led to the establishment of RDCJ. During his studies in the UK, he encountered the concept of open innovation for the first time, witnessing firsthand the dynamic interactions between patient advocacy groups and pharmaceutical companies. This collaborative spirit, where stakeholders actively exchanged ideas and worked together towards developing new therapies, left a profound impression on him.

Dr. Aoki recounted feeling a strong sense of urgency, fearing that Japan's rare disease treatment landscape might fall behind if it did not adopt similar collaborative practices. This realization became the catalyst for his efforts to establish the consortium. Upon his return to Japan, he dedicated himself to organizing workshops and building the groundwork for RDCJ, culminating in the successful launch of the consortium on this significant day.

In his presentation, Dr. Aoki underscored the importance of creating an ecosystem centered on the patient journey, recognizing that addressing the multifaceted challenges of rare diseases requires a holistic approach. He emphasized that solutions must consider the entire path patients take from diagnosis through treatment and ongoing care. Dr. Aoki highlighted the necessity of fostering cooperation among five key sectors: industry, government, academia, patients, and the community. He passionately called for the creation of a society where individuals with rare diseases can lead diverse and fulfilling lives, underpinned by a supportive and innovative ecosystem. This vision, he asserted, is crucial for Japan to maintain its competitiveness in the global arena of rare disease research and treatment.

## Key sessions and discussions

### Session 1: patient-centered healthcare services

#### Speaker: Dr. Yoshinao Mishima

Dr. Yoshinao Mishima, President of the Japan Agency for Medical Research and Development (AMED), was the first speaker in this session. Dr. Mishima provided an in-depth overview of the pivotal role of AMED in supporting research and development for rare and intractable diseases. He began by explaining that AMED, established in 2015, functions as a central funding agency in Japan, selecting outstanding research projects and researchers through competitive public calls and allocating necessary research funds. Although AMED itself does not conduct research, it plays a crucial role in facilitating and advancing medical research nationwide.

Dr. Mishima highlighted the significant contributions of AMED to the development of therapies for rare diseases, including their support for groundbreaking nucleic acid medicines like viltolarsen, a drug developed for Duchenne muscular dystrophy in collaboration with Dr. Yoshitsugu Aoki and his team at the National Institute of Neuroscience, NCNP. This exemplifies AMED's commitment to fostering innovative treatments for rare diseases.

Moreover, Dr. Mishima announced the introduction of a new support framework for ultra-orphan diseases, defined as conditions affecting fewer than 1000 patients in Japan. This initiative, launched in the previous fiscal year, aims to address the urgent need for research and development in this highly underserved area. By broadening their funding mechanisms to include ultra-rare diseases, AMED hopes to spur the creation of more novel therapies originating from Japan.

Throughout his presentation, Dr. Mishima emphasized the importance of patient-centered approaches in healthcare services. He stressed that understanding and addressing the unique needs of patients are crucial for the successful development and implementation of new treatments. The lecture concluded on an optimistic note, with heightened expectations for the emergence of new, Japan-developed drugs to meet the needs of rare disease patients.

#### Speaker: Ms. Kaori Ikegami

Ms. Kaori Ikegami, Vice President of the Japan Muscular Dystrophy Association, shared her deeply personal journey as the mother of a child with congenital muscular dystrophy. Speaking from the perspective of a family member, Ms. Ikegami illustrated the patient's journey by recounting her experiences and the daily realities faced by her and her son. Her son, now in the second year of middle school, was definitively diagnosed with congenital muscular dystrophy at just seven months old. Ms. Ikegami reflected on the overwhelming grief she felt upon receiving the diagnosis, particularly when the doctor conveyed the prognosis that her son would not live long. She admitted, “I do not know how many days I cried.”

As her son grew older, his ability to perform everyday tasks progressively declined. Currently, he is unable to move his body even a millimeter, requiring assistance to turn in bed every few hours during the night. Ms. Ikegami described the frequent interruptions to her sleep, awakened by her son's cries for help. Despite these immense challenges, she shared a heartwarming aspect of their journey: her son enjoys his days surrounded by young nurses, and she displayed photos of him smiling contentedly in their company. With a smile, she noted, “Surprisingly, he seems to be enjoying his life.”

Ms. Ikegami expressed her optimism about the establishment of the RDCJ, which aims to address the issue of drug lag in Japan. She voiced her hope that this initiative will accelerate the development and availability of treatments for rare diseases. Furthermore, she extended a heartfelt offer of support from patient advocacy groups to the industry, government, and academic representatives in attendance, urging them to reach out for any assistance they may need in the realm of rare disease drug development. Her testimony underscored the emotional and practical challenges families face and highlighted the critical need for comprehensive and supportive healthcare systems for rare disease patients.

### Video seminar: developing treatments for rare diseases - insights from familial Alzheimer's disease research

#### Speaker: Dr. Takeshi Iwatsubo

Dr. Takeshi Iwatsubo, Director General of the National Institute of Neuroscience at the NCNP and Professor of Neuropathology at the University of Tokyo's Graduate School of Medicine, presented a compelling analysis during the video seminar titled “Developing Treatments for Rare Diseases: Insights from Familial Alzheimer's Disease Research.” Dr. Iwatsubo highlighted the unexpected commonalities between dementia, which affects one in six elderly individuals, and rare diseases, which affect less than 0.1% of the population. Despite their seemingly opposite nature, both types of diseases are progressive and severe, requiring early detection and intervention to achieve maximum therapeutic effect.

He noted that recent scientific advancements have led to the development of new treatments for both conditions. For Alzheimer's disease, a significant breakthrough was achieved with a new antibody drug that successfully slowed the progression of early-stage Alzheimer's by approximately 30%.^9^ This success, Dr. Iwatsubo emphasized, was largely due to various industry-academia collaborations.

Drawing parallels to the field of rare diseases, Dr. Iwatsubo stressed the importance of strong connections among patients, pharmaceutical companies, and academic institutions. He underscored that the involvement of patients and their families in the research and development process is crucial for achieving successful outcomes. Concluding his remarks, Dr. Iwatsubo expressed his admiration for those dedicated to this mission, encouraging them to continue striving towards their goals with the collective effort of all stakeholders involved.

### Muscular dystrophy public lecture (joint session)

#### Speaker: Mr. Yasushi Okada

In the subsequent public lecture, Mr. Yasushi Okada, President of the Japan Pharmaceutical Manufacturers Association, addressed the pressing issue of drug lag, where medications approved overseas are unavailable or significantly delayed in Japan. He highlighted that this problem is particularly severe in the realm of rare diseases. Of over 200 rare disease medications approved internationally, approximately half remain unapproved in Japan.^10^ Mr. Okada identified several factors contributing to this issue: many of these medications are developed by emerging companies without a presence in Japan, and the investment in Japan's emerging biotech sector is significantly lower—by an order of magnitude—compared to that in the United States. Consequently, Japan's drug development, particularly in areas such as antibody therapies and gene treatments, is lagging.

Mr. Okada also reflected on the global race to develop COVID-19 vaccines, noting Japan's lack of significant presence and emphasizing that such outcomes should not be repeated. He called for a united effort across Japan to address these delays and bolster the country's pharmaceutical innovation ecosystem, aiming to ensure timely access to critical treatments for rare diseases.

### Towards patient-friendly wearable clinical trials

#### Speaker: Dr. Hisanobu Kaiya

Dr. Hisanobu Kaiya, Senior Advisor of the Japan Muscular Dystrophy Association, delivered a thought-provoking presentation on the development of patient-friendly wearable and decentralized clinical trials. As a psychiatrist and the father of a progressive muscular dystrophy patient, Dr. Kaiya has been deeply involved in organizational reforms during his tenure as the association's executive director emeritus. One of his key initiatives was the establishment of the Clinical Trial Research Promotion Organization in Japan.

Dr. Kaiya highlighted the significant challenges faced by muscular dystrophy patients, who often have to relocate near trial facilities to continue their participation due to the progression of their disease, which makes walking increasingly difficult. In response, the association has not only supported patient recruitment for trials but also launched independent grant activities aimed at minimizing the pain and burden on trial participants.

He emphasized the potential of alternative specimens to painful biopsies and the possibility of wearable trials that patients can participate in from home. Dr. Kaiya called for the swift realization of these technologies in muscular dystrophy trials. He urged attendees to utilize wearable trials to accelerate the development of new drugs, emphasizing the importance of innovative approaches to alleviate the hardships faced by trial participants.

### Individual session: advancing research and development in rare and intractable disease healthcare

#### Speaker: Mr. Masahiro Ishida

Following a short break, an individual session was held featuring six representatives from industry, government, and academia. Leading the session was Mr. Masahiro Ishida, leader of the Rare Disease Task Force at the Japan Pharmaceutical Manufacturers Association (JPMA). Ishida presented the findings from various surveys conducted by JPMA concerning rare diseases, shedding light on the challenges faced within Japan's rare disease landscape.

One notable survey result revealed that three out of four patients expressed a desire to learn more about their diseases, the medications they are taking, the development of new drugs, and ongoing clinical trials in Japan. However, these patients reported difficulties in obtaining such information, attributing it to the limited communication from Japanese companies. In contrast, international pharmaceutical companies were actively disseminating information online, leading many Japanese patients to seek out foreign websites for updates. This discrepancy has resulted in criticism, with patients questioning, “Why are only Japanese companies so closed off?”

Mr. Ishida explained that JPMA is preparing a set of recommendations to address these issues, with plans to release the proposal soon. He emphasized the importance of ongoing discussions to resolve the various problems identified, aiming to improve the information flow and support for rare disease patients in Japan.

### Efforts of the ministry of economy, trade, and industry in biopharmaceutical development

#### Speaker: Mr. Hiroaki Shimoda

Mr. Hiroaki Shimoda, Director of the Bio-Industry Division at the Ministry of Economy, Trade, and Industry (METI), discussed the ministry's initiatives aimed at supporting biopharmaceutical development, particularly in the field of rare diseases. Mr. Shimoda highlighted that the central role in new drug development for rare diseases has shifted from traditional mega-pharma companies to smaller entities such as academia and emerging enterprises.

Given that Japan's emerging enterprise market is significantly smaller in scale compared to its international counterparts, METI has embarked on distinct support activities to attract more investment. These initiatives differ from those of the Ministry of Education, Culture, Sports, Science, and Technology (MEXT) and the Ministry of Health, Labour, and Welfare (MHLW). For example, METI organized “Japan Innovation Night” in Boston this past June to introduce promising Japanese startups to overseas investors.

Additionally, to bolster the entire ecosystem, METI launched the “Drug Discovery Venture Ecosystem Enhancement Project” last fiscal year. Under this project, venture capitalists (VCs) approved by METI who invest in domestic startups receive supplementary funding from METI to support these companies further. Mr. Shimoda expressed his commitment to strengthening Japan's drug discovery capabilities, noting the missed opportunity during the development of COVID-19 vaccines. He emphasized the importance of these new support systems in enhancing Japan's pharmaceutical innovation and ensuring the country can contribute more effectively to global drug development efforts.

## Individual session: advancing research and development in rare and intractable disease healthcare

### Current Status and future prospects of the undiagnosed diseases initiative (IRUD)

#### Speaker: Dr. Hidehiko Mizusawa

Dr. Hidehiko Mizusawa, Special Adviser to the President and Honorary President of the NCNP, discussed the “Initiative on Rare and Undiagnosed Diseases (IRUD),” a program aimed at diagnosing patients with clinical findings that elude standard medical diagnosis through comprehensive genome analysis.^11^ As the lead for this initiative, he highlighted the nationwide network of 52 clinical and semi-clinical centers from Hokkaido to Okinawa. When patients with challenging diagnostic problems are identified, they undergo definitive diagnosis using next-generation sequencers. To date, over 20,000 individuals have undergone genetic testing, with nearly 7000 families registered in the program. Remarkably, more than 40% of these cases have resulted in definitive diagnoses. Some patients, following their diagnoses, have received treatment and are now healthy enough to attend school regularly.

However, Dr. Mizusawa noted a significant challenge: approximately half of the patients who have undergone testing remain undiagnosed. He stressed the need for expanding the initiative and sharing data with researchers both domestically and internationally to identify the genetic causes of these enigmatic diseases. He expressed a hopeful vision for the future, aiming to eventually eliminate undiagnosed diseases by uncovering their genetic underpinnings.

### Activities and prospects of the national center biobank network

#### Speaker: Dr. Yuichi Goto

Dr. Yuichi Goto, Director of the Biobank at the NCNP and Advisor for the Central Biobank at the National Center for Global Health and Medicine, provided an overview of the activities and future prospects of the National Center Biobank Network (NCBN).^12^ The NCNP runs a biobank initiative where, with patient consent, blood and tissue samples from patients with intractable diseases are collected, linked with clinical information, and stored for research purposes. These samples are then made available to disease researchers both domestically and internationally. For instance, in the case of muscular dystrophy, muscle tissues and muscle-derived culture cells collected from patients for diagnostic purposes are cryopreserved for future research use.

This biobank initiative has already yielded significant research outcomes. One notable success is the contribution to the understanding and treatment of mitochondrial diseases, specifically mitochondrial myopathy, encephalopathy, lactic acidosis, and stroke-like episodes (MELAS). This designated intractable disease has led to the development of a taurine supplementation therapy. Dr. Goto emphasized the biobank's critical role in bridging clinical practice and research, and expressed his commitment to continuing and expanding the biobank initiative. He highlighted the importance of the biobank in facilitating ongoing and future research, which is essential for advancing medical knowledge and treatment options for rare diseases.

### Individual session: advancing research and development in rare and intractable disease healthcare

#### Panel discussion

The session concluded with a comprehensive panel discussion featuring Mr. Masahiro Ishida, Dr. Shun Tezuka, Mr. Jiro Ezaki, Dr. Hidehiko Mizusawa, and Dr. Yuichi Goto. A key issue addressed was the lack of accessible information for patients. Mr. Jiro Ezaki from the Ministry of Health's Rare Disease Control Division noted that many patients report insufficient explanations from their primary doctors regarding medical expense subsidies and social welfare systems. Dr. Yuichi Goto shared an instance where a consultation service on a rare disease association's website had to be temporarily closed due to an overwhelming number of personal inquiries from patients. He highlighted the problem that patients have many questions but do not know whom to ask, nor do they have anyone to confide in about their concerns. Dr. Hidehiko Mizusawa pointed out that while there is a significant amount of information available online, the issue is the lack of clear, understandable information. Mr. Masahiro Ishida agreed, noting that the abundance of information can be overwhelming, making it difficult for patients to discern what is accurate. He admitted that even he finds clinical trial information complex and hard to understand. Dr. Shun Tezuka introduced the PMDA's email newsletter service (PMDA Medi-Navi) and encouraged its use as a resource for reliable information.^13^ The panel underscored the need for better communication channels and more accessible, clear information to help patients navigate their healthcare journeys effectively.

### Keynote address: antisense oligonucleotide therapeutics - opportunities for rare brain disorders

#### Speaker: Dr. Annemieke Aartsma-Rus

Dr. Annemieke Aartsma-Rus, a professor at Leiden University Medical Center, delivered a compelling keynote address on the opportunities for antisense oligonucleotide (ASO) therapies in treating rare brain disorders. She represents the “N = 1 Collaborative”, a global network of researchers dedicated to the practical application of personalized medicine for rare diseases.^14^

Dr. Aartsma-Rus began by sharing the poignant story of a seven-year-old girl named Mila, whose case was the catalyst for the formation of the network. Mila suffered from a fatal neurogenetic disorder, and in a remarkable collaborative effort, scientists, doctors, foundations, and regulatory agencies developed a personalized treatment for her within just one year. This treatment, named Milasen in her honor, significantly alleviated her symptoms, including seizure frequency, thereby improving her quality of life.^15^ Sadly, despite these efforts, Mila's disease had progressed too far by the time treatment was started, and she passed away in 2021.^16^

This case, however, provided invaluable insights and inspiration for many researchers, including Dr. Aartsma-Rus, who has been researching nucleic acid medicines for muscular dystrophy. The success and limitations of Milasen underscored the potential and challenges of personalized medicine in rare diseases. Driven by this experience, Dr. Aartsma-Rus helped establish the Dutch Center for RNA Therapeutics and the “N = 1 Collaborative,” national and international networks, respectively, aiming to develop optimized treatments tailored to individual patients with rare diseases.

In her lecture, Dr. Aartsma-Rus acknowledged the inherent uncertainties and challenges in developing these therapies, emphasizing that perfection cannot be achieved from the outset. She highlighted the importance of perseverance and iterative improvement in the field. Dr. Aartsma-Rus concluded with a hopeful vision for the future, expressing her commitment to continuing the development of personalized therapies for rare disease patients worldwide.

## Closing remarks

The symposium concluded with insightful addresses from the distinguished members of the RDCJ Organizing Committee.

Dr. Yoshitsugu Aoki, Head of the Department of Gene Therapy at the National Center of Neurology and Psychiatry: Dr. Aoki opened by expressing his gratitude to all participants, emphasizing his long-standing dedication to treating diseases. He recalled his early motivations as a physician and how encountering rare diseases intensified his resolve to find solutions. Dr. Aoki shared that the symposium had reignited his commitment to translating research into therapies for rare and intractable diseases. He reflected on the invaluable learning experiences from meeting and conversing with various stakeholders, which broadened his previously narrow perspective. Dr. Aoki highlighted the critical juncture at which his research stands and urged all attendees to support these vital efforts in developing pharmaceuticals for rare diseases.

Dr. Toshifumi Yokota, Professor at the University of Alberta: Dr. Yokota, leading a research lab in Canada as the Muscular Dystrophy Canada research chair for over a decade, underscored the critical importance of international collaboration in the fight against rare diseases. Emphasizing the significant role patient organizations play in developing new treatments, such as gene therapy and antisense drugs, Dr. Yokota acknowledged the differing approaches between Canada and Japan. However, the RDCJ was highlighted as a promising bridge for international cooperation, crucial for overcoming challenges such as limited patient numbers, experts, and resources in each country. Optimism was expressed that the consortium will facilitate meaningful partnerships with various global entities, including patient and private organizations, to advance research and treatment.

Dr. Naoto Inukai, Director at Takeda Pharmaceutical Co. Ltd.: Representing the corporate members, Dr. Inukai thanked all participants for their engagement. He highlighted the collaborative spirit of the symposium, noting that while pharmaceutical companies often see each other as competitors, in this context, they are united against the common adversary of rare diseases. Dr. Inukai emphasized that this spirit of camaraderie extends beyond the pharmaceutical industry to include academia, government, patients, and their families. He called on all stakeholders to continue supporting the RDCJ's mission and consider joining its efforts to overcome the challenges of treating rare diseases.

Dr. Hiroyuki Shibasaki, Japan Muscular Dystrophy Association: Dr. Shibasaki shared his personal journey, having been diagnosed with muscular dystrophy 22 years ago, and how this experience shaped his involvement in the rare disease community. He reflected on the significant advancements in the diagnosis and treatment of muscular dystrophy over the past two decades, attributing these successes to collective effort and collaboration. On the other hand, he also pointed out that there is still a lack of its fundamental treatments. Therefore, Dr. Shibasaki encouraged symposium participants to leverage the connections made during the event to pursue the goal of developing and delivering treatments for rare diseases. He reminded everyone that the symposium's success is merely the starting point and urged them to remain committed to the ongoing work required to make a tangible impact.

## Conclusion

The inaugural symposium of the Rare Disease Consortium of Japan (RDCJ) successfully brought together diverse stakeholders to address the multifaceted challenges of rare diseases. The event underscored the necessity of a collaborative approach and set the stage for future initiatives aimed at improving the lives of those affected by rare diseases in Japan. As participants celebrated the achievements of the symposium, they were encouraged to stay invigorated and ready to push forward, turning today's discussions into tomorrow's advancements.
